# Initial disease severity and quality of care of emergency department sepsis patients who are older or younger than 70 years of age

**DOI:** 10.1371/journal.pone.0185214

**Published:** 2017-09-25

**Authors:** Mats Warmerdam, Frank Stolwijk, Anjelica Boogert, Meera Sharma, Lisa Tetteroo, Jacinta Lucke, Simon Mooijaart, Annemieke Ansems, Laura Esteve Cuevas, Douwe Rijpsma, Bas de Groot

**Affiliations:** 1 Emergency Department, Leiden University Medical Centre, Leiden, Zuid-Holland, the Netherlands; 2 Department of Gerontology and Geriatrics, Leiden University Medical Centre, Leiden, Zuid-Holland, The Netherlands & Institute for Evidence-based Medicine in Old Age | IEMO, Leiden, The Netherlands; 3 Emergency Department, Albert Schweitzer Ziekenhuis, Dordrecht, Zuid-Holland, the Netherlands; 4 Emergency Department, Rijnstate Ziekenhuis, Arnhem, Gelderland, the Netherlands; Azienda Ospedaliero Universitaria Careggi, ITALY

## Abstract

**Objective:**

Due to atypical symptom presentation older patients are more prone to delayed sepsis recognition. We investigated whether initial disease severity *before* emergency department (ED) treatment (including treatable acute organ dysfunction), quality of ED sepsis care and the impact on mortality was different between patients older and younger than 70 years. If differences exist, improvements are needed for ED management of older patients at risk for sepsis.

**Methods:**

In this observational multicenter study, ED patients who were hospitalized with a suspected infection were stratified by age <70 and ≥70 years. The presence of treatable and potentially reversible acute organ dysfunction was measured by the RO components of the Predisposition, Infection, Response and Organ dysfunction (PIRO) score, reflecting acute sepsis-related organ dysfunction developed *before* ED presentation. Quality of care, as assessed by the full compliance with nine quality performance measures and the standardized mortality ratio (SMR: observed/expected in-hospital mortality), was compared between older and younger patients.

**Results:**

The RO-components of the PIRO score were 8 (interquartile range; 4–9) in the 833 older patients, twice as high as the 4 (2–8; P<0.001) in the 1537 younger patients. However, full compliance with all nine quality performance measures was achieved in 34.2 (31.0–37.4)% of the older patients, not higher than the 33.0 (30.7–35.4)% in younger patients (P = 0.640). In-hospital mortality was 9.2% (95%-CI, 7.3–11.2) in patients ≥70, twice as high as the 4.6% (3.6–5.6) in patients <70 years, resulting in an SMR (in study period) of ~0.7 in both groups (P>0.05).

**Conclusion:**

Older sepsis patients are sicker at ED presentation but are not treated more expediently or reliably despite their extra acuity The presence of twice as much treatable acute organ dysfunction *before* ED treatment suggests that acute organ dysfunction is recognized relatively late by general practitioners or patients in the out of hospital setting.

## Introduction

### Background

Sepsis is defined as life-threatening organ dysfunction caused by a dysregulated host response to infection, according to the sepsis-3 definition [1]. The incidence of sepsis increases with age due to increasing comorbidity, exposure to instrumentation, institutionalization, immune-senescence and malnutrition [2–4], while the outcome of older patients with sepsis is worse compared to younger patients, which is associated with higher health-care costs [2–5].

Early sepsis recognition and quality of emergency department (ED) sepsis care is therefore important to improve outcome of older patients [2, 6, 7]. However, atypical symptom presentation, including delirium, malaise and functional decline, and the absence of classical symptoms such as fever, tachycardia and hypoxemia may result in poor sepsis recognition and delayed presentation to a doctor. As a result, older patients may present to the ED with more acute potentially reversible sepsis-related organ dysfunction, especially because older patients have a higher risk for deterioration due to less physiological reserve [2].

In addition, poor sepsis recognition may also affect the quality of care of older patients *in* the ED which has a large impact on mortality. In a recent study, implementing a “Surviving Sepsis Campaign” based quality improvement program, it was shown that in patients who were hospitalized with a suspected infection, full compliance with nine quality performance measures was associated with a large reduction of in-hospital mortality [8]. Unfortunately, full bundle compliance was achieved in only ~40% of the patients. Previous studies suggest that this may be even worse in older patients due to the aforementioned poor sepsis recognition [2, 5, 9–11].

### Importance

If quality of ED sepsis care is worse in older patients with sepsis, i.e. due to poor recognition of acute sepsis-related organ dysfunction, quality improvement programs should put more emphasis on optimization of sepsis recognition and treatment of older patients in the ED as well as in the out of setting. Because many older ED patients will have a “Do Not Resuscitate (DNR)” code, and will consequently not be admitted to an intensive care unit (ICU), treatment optimization should take place in the ED setting.

### Goals

Therefore, the aim of the present study was to assess if disease severity and quality of ED sepsis care and its impact on mortality, differs between patients younger and older than 70 years of age.

## Materials and methods

### Study design and setting

This is an observational multicenter study using an existing database in which data were and are still prospectively collected as part of an ongoing quality improvement program. In the Leiden University Medical Centre, a tertiary care center with an annual census of ~30,000 patients, data were collected from 1 April 2011 to 1 February 2016. In the Rijnstate Hospital, an urban care center with ~30,000 ED visits per year, data were collected from 1 March 2012 to 1 November 2012. Recently, the Albert Schweitzer Hospital (ASZ), an urban care facility with an annual census of ~25,000 also started participation in the quality improvement program. In the ASZ data were collected from 1 September 2015 to 1 December 2015.

### Selection of participants

All consecutive ED patients > 16 years old with a suspected infection and Manchester triage category yellow, orange or red (constituting those with urgent medical needs) [12] who received intravenous antibiotics in the ED and were subsequently hospitalized were included in the database. After inclusion in the database, patients were stratified in a group of 70 years and older and a group younger than 70 years.

The study was approved by the medical ethics committee of the Leiden University Medical Center, who waived the need for individual informed consent as this was a purely observational study.

### Sepsis quality improvement program

The sepsis quality improvement program included a standard screening procedure aimed at facilitating sepsis recognition (including clinical and biochemical signs of acute organ dysfunction), early ED resuscitation and disposition to an appropriate level of care (S1 Fig).

The screening procedure was developed by an expert group of ED physicians, intensivists, surgeons and infectious disease specialists from the Leiden University Medical Centre and was based on the “Surviving Sepsis Campaign” sepsis program of the National Patient Safety Agency (Safety Management System [VMS]), which had been strongly promoted at the hospitals by means of workshops, presentations and posters (for details see: http://links.lww.com/CCM/A923). The identical quality improvement program was voluntarily implemented in the participating hospitals at different time points. The program was not funded by the hospitals or an external group as a specific initiative.

Two emergency physicians (BdG, AA) informed all ED nurses, physicians, residents and staff of all ED specialties about the program, the data that would be collected as part of the program, and the assessment of the program through oral presentations, posters and flyers in the ED. The same two emergency physicians instructed new medical personnel who missed the official launch of the program.

ED patient entry into the sepsis quality improvement program began at triage at ED presentation or at any time during their ED evaluation. Patients with symptoms or signs of suspected infection (e.g. fever, coughing or erythema) triggered the initiation of sepsis screening by the triage nurses or the nurses or physicians caring for the patients during their ED stay. The triage or treating nurse placed a patient sticker on the patient registration form of patients with a suspected infection and whose triage category was yellow, orange or red. From that moment, nurses and doctors had to follow the protocol and the collection of data started.

### Methods and measurements

The data collected included demographic characteristics, patients’ co-morbidity, time points of start of oxygen and fluid therapy and antibiotic administration, laboratory values, triage categories and vital signs, treatment administered (including antibiotics, intravenous fluids and oxygen), disposition from the ED and measured outcomes. The suspected source of infection in the conclusion of the medical file of the treating physician at the time of ED presentation was also registered. We did not use ICD-10 codes.

Data were prospectively registered in the digital hospital information system (Chipsoft, Amsterdam, Netherlands).

A medical student or registrar in emergency medicine subsequently transferred data from the electronic hospital information system to a web-based data collection file (Promise, Leiden, Netherlands), which automatically calculated the illness severity scores. In addition, to limit typing errors, Promise warned if entered variables had extreme or illogical values. (For detailed information about Promise see https://www.msbi.nl/promise/promise.aspx.) Each month BdG and AA checked whether the medical students or residents still complied with the definitions of quality performance measures.

After the inclusion period, data of the three participating hospitals were downloaded to one SPSS file (SPSS version 23.0, IBM, New York, USA) and the data were analyzed as described below.

#### Disease severity

The following measurements or calculations represent disease severity and were carried out during patient follow-up because the results were not yet known during ED presentation:

Firstly, disease severity was assessed using the patient’s initial Predisposition, Infection, Response and Organ dysfunction (PIRO) score. As described previously [13, 14], the PIRO score, which has previously been validated for use in the Netherlands’ ED setting, was used because this measurement enabled separation of the non-modifiable predisposition and infection aspects (the PI components) of disease severity from the potentially treatable response and acute organ dysfunction aspects of disease severity (RO components). This RO component is distinctly different from chronic organ dysfunction. Missing values used to calculate the PIRO score were imputed as normal values by Promise, as was done previously in the Acute Physiology and Chronic Health Evaluation (APACHE) score calculations [15].

Secondly, organ dysfunction was also calculated according to the definition of Dellinger et al. [6].

Thirdly disease severity was determined by the percentage of sepsis patients that was transferred from the ED to the ICU, corrected for “do not resuscitate” (DNR) status, because these patients were not eligible for an ICU transfer. A patient was considered to have a DNR status if existing medical files already stated that the patient had a DNR status or when it was decided at the time of ED presentation or during hospital admission.

#### Quality of care

Quality of care was assessed in two ways: firstly, by assessment of the number of achieved quality performance measures (as described below), or by the percentage of patients in whom all nine quality performance measures were achieved (full compliance). The rationale behind and the impact of the nine quality performance measures used in the present study have been described in detail previously [8]. Secondly, quality of care will be calculated by the standardized mortality ratio (SMR: for definition see below).

For each individual patient the following 9 quality performance measures were assessed:

First, administration of antibiotics within 3 hours after ED registration. Time to antibiotics was measured by subtraction of registration time at the ED desk from the registered time of antibiotic administration by the nurse [16–18].

Second, the appropriateness of the initial dose of antibiotics administered in the ED was assessed in retrospect as described previously (S2 Fig) [19, 20].

Third, obtainment of blood cultures before administration of antibiotics [6].

Fourth, the accuracy of the suspected source of infection was assessed by comparison with the suspected source of infection in the final hospital discharge letter, which was considered the “gold standard” with regard to accuracy of diagnosis. If the initial suspected source of infection differed from the final hospital discharge letter, the initial ED working diagnosis was considered to be incorrect.

Fifth, lactate measurement <6 hrs after ED registration [6].

Sixth, mean arterial pressure >65 mmHg <6 hours after ED registration [6].

Seventh, ICU consultation in the case of severe sepsis or septic shock. In the Netherlands, treatment requiring central venous and arterial catheters is usually performed in the ICU and not in the ED. Optimal hemodynamic resuscitation therefore requires a consultation with the ICU. Early transport to an optimal level of care has been shown to improve outcome [21].

Eighth, administration of at least 1.5 L of fluids in the case of shock, in accordance with the SSC recommendations [6]. Any amount of fluids was considered “sufficient” as long as an ED patient did not have signs of shock, i.e. lactate >4 mmol/L and/or systolic blood pressure <90 mmHg or a decrease of the systolic blood pressure of > 40 mmHg compared to the baseline blood pressure of the ED patient.

Ninth, *no* unanticipated transfer from ward to ICU. A patient was considered to have an unanticipated transfer from the ward to the ICU if he/she was first admitted to a normal ward and had a subsequent transfer to the ICU <48 hours after hospital admission because of sepsis progression. In previous studies it has been shown that unanticipated transfers from a ward to the ICU have a negative impact on outcome [21–23]. Although the result of the disposition decision is only known in retrospect, it is a reflection of the recognition and adequate interpretation of the severity of the illness, and the response to ED treatment *in* the ED. This is facilitated by the ED sepsis algorithm (S1 Fig). This performance measure was considered to be achieved if there was *no* unanticipated transfer from the ward to the ICU.

A second measure we used to quantify overall quality of care was the SMR, the observed in-hospital mortality divided by the expected mortality, i.e. predicted mortality calculated with the PIRO score [24, 25]. Five PIRO categories (0–4, 5–9, 10–14, 15–19, >20) were defined and associated with observed mortality. We calculated the PIRO score in our study for each individual patient and assigned an expected mortality based on the mean observed mortality in original study by Howell et al ([13] fig 3). For the groups younger and older than 70 years, the mean expected mortality was calculated (in %). The observed percentage of in-hospital mortality was calculated by dividing the observed number of deaths by the total number of patients in the groups younger or older than 70 years. This sepsis-specific SMR provides an overall measure for quality of care which is independent of changing insights of effectivity of quality performance measures. More importantly, it takes into account potential quality performance measures which are currently unknown or unmeasurable.

### Outcomes

This study had two outcome measures; First, the presence of treatable acute organ dysfunction, as assessed by the RO components of the PIRO score, reflecting organ dysfunction developed before ED presentation. Secondly, quality of care as assessed by the percentage of patients in whom full compliance with all 9 quality performance measures were achieved, and the SMR.

### Data analysis

#### Statistical analyses

Screening and enrolment results were summarized. Patient characteristics were described as follows: normally distributed data were presented as mean (standard deviation: SD) and skewed data as median (interquartile range: IQR). Categorical data were presented as percentage of total.

Differences of disease severity between younger and older patients were tested with independent Student t-tests or Mann Whitney U tests, as appropriate.

Differences between younger and older patients of percentage ICU admissions, percentage of patients with full compliance with (all nine) quality performance, and percentage of the individual achieved quality performance measures were tested with the chi-square test.

We subsequently assessed the impact on in-hospital mortality of the achievement of *all* performance measures using multivariable binary logistic regression analysis, adjusting for the PIRO score (categorized as 0–8, 9–17 and > 17), disposition location (to ward or ICU) and hospital (University medical center of peripheral hospital), which were all forced into the model, similarly as described previously [8].

Before the prediction model was developed, we first tested whether the association between full compliance with all 9 quality performance measures and in-hospital mortality differed between ED patients older and younger than 70 years of age by the addition of an interaction term. A non-significant interaction term would indicate that one prediction model should be developed for all included patients.

The PIRO score reflects disease severity and consists of 16 variables, including demographic data, type of infection, vital signs and biochemical signs of organ dysfunction. To prevent multicollinearity, these individual variables were not put into the prediction model together with the total PIRO score. In addition, disposition was also forced into the model because this variable partially reflects response to ED treatment (which reflects a different aspect of disease severity) and partially reflects the quality of management on the ward or ICU [14].

Hosmer and Lemeshow’s goodness-of-model-fit testing was used to assess model fitness. Receiver operator characteristics with area under the curve (AUC) analyses (c-statistic) was used to assess the discriminative performance of the model.

Variance inflation factors (VIFs) were used to investigate multicollinearity. If the VIF was below 3, multicollinearity was not considered to be a problem. Odds ratios (ORs) and percentage of patients in whom a quality performance measure was achieved was reported with 95% confidence intervals (95%-CIs). The 95% confidence interval of the SMR was calculated using an online calculator (http://www.openepi.com/SMR/SMR.htm).

For all analyses, an α of 0.05 was used to assess statistically significant differences.

All data were analyzed using SPSS software (SPSS 23.0, IBM, New York, USA).

#### Sample size estimates

Based on a previous study in our hospital [8], we expected that full compliance to all quality performance measures would be achieved in ~40%. To be able to detect a difference in full bundle compliance of 40% in the younger group and 30% in the older group with a power of 80% and a significance level of 5% at least 356 patients were needed in each group.

For the multivariable logistic regression analysis we used the rule of thumb that approximately 10 events per covariate were needed. Because we wanted to put at least 6 variables in the model, 60 events were needed. Hence, the database we used contained more than enough patients.

## Results

### Characteristics of study subjects

**Fig 1:**
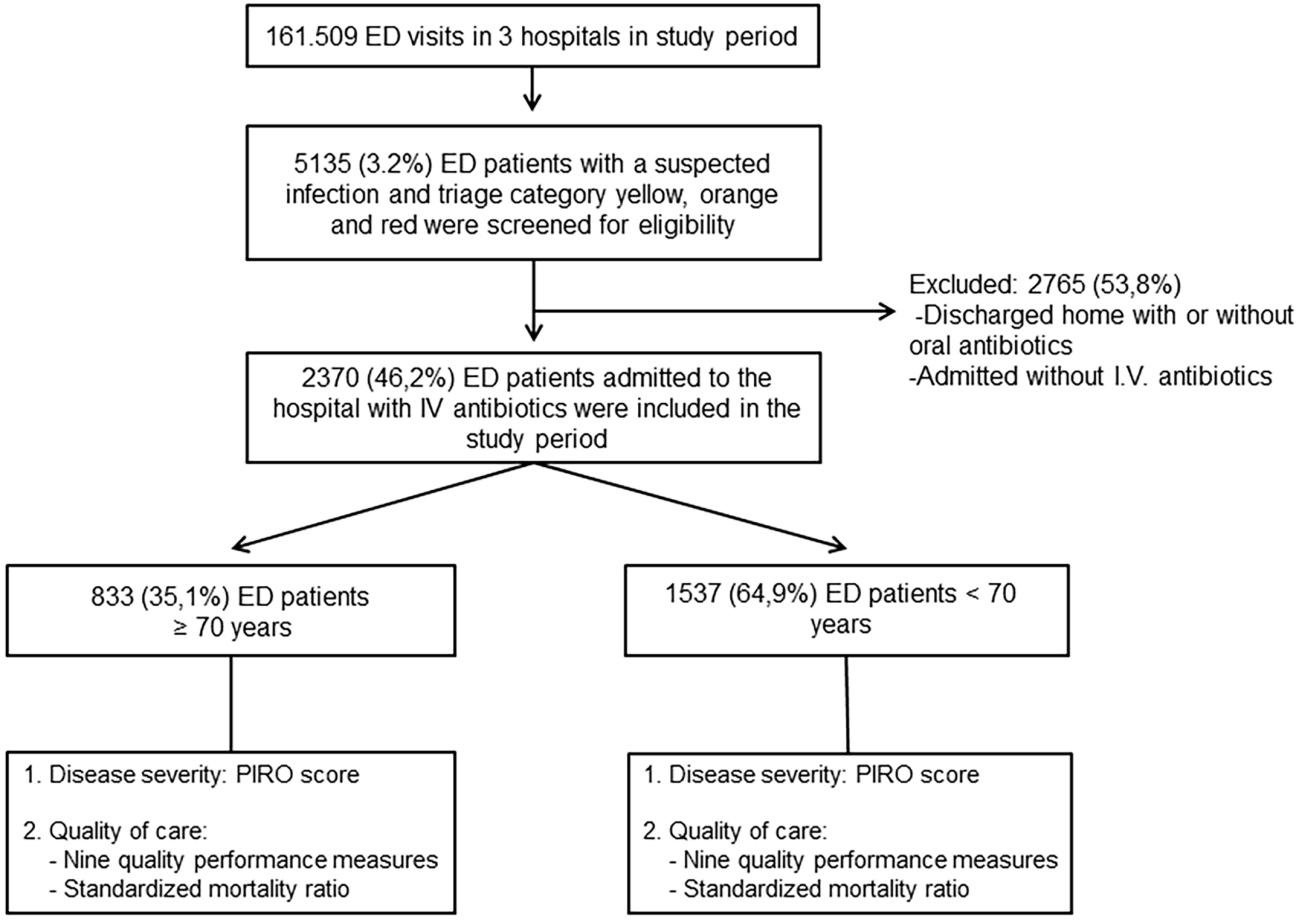
Patient inclusion and flow through study.

Fig 1 shows the patient flow through the study; 833 of the 2370 included patients were 70 years and older (35.1%), and 1537 patients were younger than 70 years (64.9%).

Table 1 shows the patient characteristics of the total group, and the groups older and younger than 70 years. The mean age in the older group was 78.4 (6.3) and 52.2 (13.6) in the younger group. 520 patients (62.4%) in the older group were men, compared to 844 patients (54.9%) in the younger group. Older patients presented more often with an altered mental status, a higher systolic blood pressure, a higher respiratory rate, and a lower heart rate.

**Table 1:** Patient characteristics of patients in groups younger and older than 70 years.

	Total cohort	<70 years	≥70 years
**Demographics**			
N (%)	2370	1537 (64.9)	833 (35.1)
Age, mean (SD)	61.4 (17.0)	52.2 (13.6)	78.4 (6.3)
Gender (male), n (%)	1364 (57.6)	844 (54.9)	520 (62.4)
Included at University Medical Centre, n (%)	1867 (78.8)	1277 (83.1)	590 (70.8)
**Co-morbidities, n (%)**			
COPD (2)	368 (15.5)	163 (10.6)	205 (24.6)
Heart failure (1)	361 (15.2)	139 (9.0)	222 (26.7)
Liver disease (1)	115 (4.9)	94 (6.1)	21 (2.5)
Renal disease (1)	435 (18.4)	267 (17.4)	168 (20.2)
Nursing home (2)	144 (6.1)	48 (3.1)	96 (11.5)
Immune-compromised (2)	971 (41.0)	733 (47.7)	238 (28.6)
Malignancy–(1) *	255 (10.8)	156 (10.1)	99 (11.9)
Malignancy + (3) *	358 (15.1)	252 (16.4)	106 (12.7)
**DNR status (%) (6)**	513 (21.6)	191 (12.4)	322 (38.7)
**Suspected source of infection, n (%)**			
Pulmonary	1117 (47.1)	642 (41.8)	475 (57.0)
Urogenital	706 (29.8)	410 (26.7)	296 (35.5)
Abdominal	411 (17.3)	280 (18.2)	131 (15.7)
Skin	210 (8.9)	151 (9.8)	59 (7.1)
Neurological	52 (2.2)	36 (2.3)	16 (1.9)
other	404 (17.0)	306 (19.9)	98 (11.8)
**Vital signs on admission**			
Systolic blood pressure, mean (SD) (244)	131.9 (25.9)	129.7 (23.5)	135.6 (29.3)
Heart rate, mean (SD) (46)	107.7 (20.2)	110.0 (19.6)	103.5 (20.6)
Respiratory rate, mean (SD) (562)	24.0 (7.2)	22.9 (6.9)	25.7 (7.4)
Oxygen saturation, mean (SD) (73)	95.2 (4.8)	95.7 (4.6)	94.1 (5.1)
Temperature (°C), mean (SD) (95)	38.72 (1.10)	38.74 (1.06)	38.67 (1.19)
Altered mental status n (%) (420)	391 (16.5)	165 (10.7)	226 (27.1)
**Laboratory analysis on admission**			
Lactate (mmol/l), median (IQR) (298)	1.9 (1.4–2.6)	1.8 (1.4–2.6)	2.0 (1.5–2.8)
Platelets (x10^9^/l), median (IQR) (41)	210 (152–281)	207 (148–280)	214 (159–284)
INR median (IQR), (765)	1.1 (1.0–1.4)	1.1 (1.0–1.3)	1.2 (1.0–2.5)
Creatinine (μ g/l), median (IQR) (13)	87 (67–120)	83 (64–111)	95 (73–134)
Urea (mmol/l), median (IQR) (38)	7.0 (5.1–10.2)	6.1 (4.6–8.8)	8.7 (6.6–12.5)
Bilirubin (μmol/L), median (IQR) (348)	12 (8–18)	12 (8–18)	12 (9–20)

Abbreviations: COPD = chronic obstructive pulmonary disease, DNR = do not resuscitate order, °C = degrees Celsius, ED = emergency department, SD = standard deviation, IQR = interquartile range.

The number between brackets indicates the amount of missing variables.

* Malignancy– = without metastases, malignancy + = with metastases.

### Disease severity

Table 2 reveals that older patients present with a higher disease severity to the ED, as indicated by the higher PIRO-score of 13 (9–16) in older patients compared to 8 (4–12) in younger patients (P = <0.001). More importantly, the potentially reversible Response and Organ dysfunction score is 8 (4–9) in older patients, twice as high as the 4 (2–8) in younger patients (P = <0.001). After adjustment for DNR status, older patients were admitted more often to the ICU (P = 0.005).

**Table 2:** Comparison of disease severity at ED presentation (before ED treatment) between patients <70 and patients ≥70 years of age.

Disease severity	Total	Age <70	Age ≥70	p-value
Total PIRO score, median (IQR)	10 (5–14)	8 (4–12)	13 (9–16)	<0.001
Total Predisposition (P) and Infection (I) score, median (IQR)	4 (2–6)	4 (2–5)	6 (4–7)	<0.001
Total Response (R) and Organ dysfunction (O) score, median (IQR)	6 (2–8)	4 (2–8)	8 (4–9)	<0.001
Total Response (R), median (IQR)	3 (0–3)	2 (0–3)	3 (0–3)	<0.001
Total Organ dysfunction (O), median (IQR)	3 (2–5)	3 (0–5)	5 (2–5)	<0.001
Number of acute organs dysfunction, median (IQR) *(1)	0 (0–1)	0 (0–0)	0 (0–1)	<0.001
Admission to ICU from ED, n (%) (12)	227 (9.6)	140 (9.1)	87 (10.4)	0.321
Admission to ICU corrected for DNR status, n (%)** (9)	181 (9.8)	115 (8.6)	66 (13.1)	0.005
Hospital lengths of stay	5 (3–9)	5 (3–8)	6 (3–10)	<0.001

Before ED management had been started, older ED patients are almost twice as ill than younger ED patients, as indicated by the higher Predisposition (P) Infection (I), Response (R) and Organ dysfunction (O) scores. Interestingly, not only the non-modifiable P and I components of the PIRO score were higher, but also the potentially modifiable R and O components. In addition older patients more frequently need an admission to ICU.

Abbreviations: PIRO = predisposition, infection, response, organ dysfunction illness severity score, IQR = interquartile range, ICU = Intensive Care Unit, DNR = Do not resuscitate.

The number between brackets indicates the amount of missing variables.

*According to Dellinger 2004 [6]

**Patients with a DNR status are not included in this calculation

### Quality of care

First, we investigated quality of care in terms of compliance with quality performance measures. The higher Response and Organ dysfunction score underlines the importance of quality of ED sepsis care in older patients. In Table 3, it is shown that quality of care in terms of number of achieved quality performance measures is similar in younger and older patients, except for minor differences in time to antibiotics administration, lactate measurement within 6 hours and mean arterial pressure (MAP) ≥65mmHg within 6 hours. Full bundle compliance with all nine quality performance measures, was ~34%, also similar in both groups (P = 0.640).

**Table 3:** Quality of care as assessed by achievement of quality performance measures in the groups <70 and ≥70 years of age.

Quality performance measure	<70 years	≥70 years	P-value
1. Antibiotics administered within 3 hours, % (24)	76.0 (73.9–78.1)	81.5 (78.9–84.1)	0.001
2. Appropriate antibiotics given, % (65)	78.0 (75.9–80.1)	77.1 (74.2–80.0)	0.568
3. Lactate measured within 6 hours, %	86.3 (84.6–88.0)	89.4 (87.3–91.5)	0.035
4. Blood cultures drawn before antibiotics administration, % (6)	95.0 (93.9–96.1)	94.2 (92.6–95.8)	0.727
5. Mean arterial pressure ≥65mmHg within 6 hours, % (28)	92.8 (91.5–94.1)	91.7 (89.8–93.6)	0.028
6. Sufficient fluid administration, % *- Amount of fluid administration (L)- Amount of oxygen supply, median (L/min) (87)	95.3 (94.2–96.4) 1 (0.5–1.5) 2 (0–5)	93.9 (92.3–95.5) 1 (0.5–1.5) 3 (2–6)	0.181 0.409 <0.001
7. Necessary consultation with ICU attending, % (3)	88.4 (86.8–90.0)	88.5 (86.3–90.7)	0.800
8. Correct suspected source of infection, % (19)	83.5 (81.6–85.4)	84.2 (81.7–86.7)	0.465
9. NO unanticipated transfer from ward to ICU, % (60)	93.3 (92.1–94.5)	93.6 (91.9–95.3)	0.424
All quality performance measures attained (full compliance), % (168)	33.0 (30.7–35.4)	34.2 (31.0–37.4)	0.640

Quality of care is similar in younger and older patients, except for minor differences in time to antibiotics administration, lactate measurement within 6 hours and mean arterial pressure (MAP).

Abbreviations: ICU = intensive care unit, L = litre.

For exact definition of quality performance measures see text.

The number between brackets indicates the amount of missing variables.

*Sufficient fluid administration was scored according to the SSC recommendations, which is administration of at least 1.5 L of fluids in the case of shock [6]. Any amount of fluids was considered “sufficient” as long as an ED patient did not have signs of shock, i.e. lactate >4 mmol/L and/or systolic blood pressure <90 mmHg or a decrease of the systolic blood pressure of > 40 mmHg compared to the baseline blood pressure of the ED patient.

Correspondent to a previous study [8], Table 4 shows that full compliance with all nine quality performance measures is an independent predictor of survival, as indicated by the adjusted OR of 0.59 (95%CI = 0.39–0.90). The non-significant interaction term of P = 0.788 shows that the association between full compliance with all nine quality performance measures and in-hospital mortality was similar between younger and older ED patients. In ED patients older and younger than 70 years there was no significant association between the PIRO score categories and full compliance with all nine quality performance measures (see Tables in S1 Tables).

**Table 4:** Multivariable logistic regression analysis for in-hospital mortality.

Variables	P-value	Adjusted OR (95% CI)
PIRO 0–8	<0.001	Reference
PIRO 9–17	<0.001	3.18 (1.87–5.41)
PIRO ≥18	<0.001	9.50 (5.01–18.03)
All quality performance measures attained (full compliance)	0.019	0.59 (0.39–0.90)
Admission to ICU and/or MCU	<0.001	4.44 (2.95–6.70)
Treatment at academic medical center	0.896	1.03 (0.67–1.58)

Correspondent to a previous study [8], full compliance with all nine quality performance measures significantly reduces the odds of mortality. The Hosmer-Lemeshow goodness-of-fit test P-value = 0.966. N = 2370. The AUC or c-statistic of the model was 0.758 (0.714–0.802) This is similar in patients younger and older than 70 years, as indicated by a non-significant interaction term between full bundle compliancy by age (P-value = 0.788). Nevertheless if we created models for younger and older patients separately, the Hosmer-Lemeshow goodness of fit test had a P value of 0.974 in older patients and 0.806 in younger patients. For the older group the AUC (or c-statistic (95%-CI)) was 0.684 (0.614–0.754). For the younger group the AUC of the model was 0.816 (0.763–0.869). (Tables in S2 Tables)

Abbreviations: PIRO = predisposition, infection, response and organ dysfunction illness severity score. ICU = intensive care unit. MCU = medium care unit. OR = odds ratio. CI = confidence interval. Full compliance to all nine quality performance measures (9/9) was compared to incomplete compliance (≠ 9/9). For exact definition of quality performance measures see text.

For all variables ORs with 95% CIs are shown.

Secondly, we quantified quality of care in terms of the sepsis specific standardized mortality ratio.

The observed in-hospital mortality of older ED patients was 77, (9.2%, 95%-CI: 7.3–11.2), twice as high as the 71 (4.6%, 95%-CI: 3.6–5.6) in younger patients (P = <0.001), respectively. Because the expected mortality was also twice as high in older compared to younger patients, the SMR was 0.68 (0.53–0.85) in younger patients, similar to the SMR of 0.69 (0.54–0.85) in older patients (P>0.05).

## Discussion

The main conclusion of this study is that older sepsis patients are sicker at ED presentation but are not treated more expediently or reliably despite their extra acuity. The presence of twice as much treatable acute organ dysfunction before ED treatment suggests that acute organ dysfunction is recognized relatively late by general practitioners or patients in the out of hospital setting.

The present study shows that at ED presentation, before ED management had been started, older ED patients are twice more ill than younger ED patients, as indicated by the higher PIRO scores. Importantly, not only the non-modifiable P and I components of the PIRO score were higher, which was expected, but also the R and O components, reflecting potentially modifiable acute organ dysfunction. To the best of our knowledge, this is the first study showing that older ED patients present to the ED with almost two times as much potentially reversible acute sepsis-related organ dysfunction. This finding corresponds with previous literature demonstrating that older patients possess more risk factors for deterioration to severe sepsis than younger patients, due to a diminished cardiopulmonary reserve, a general decrease in organ function and an often enhanced innate immune response with increased cytokine production [2].

**Fig 2:**
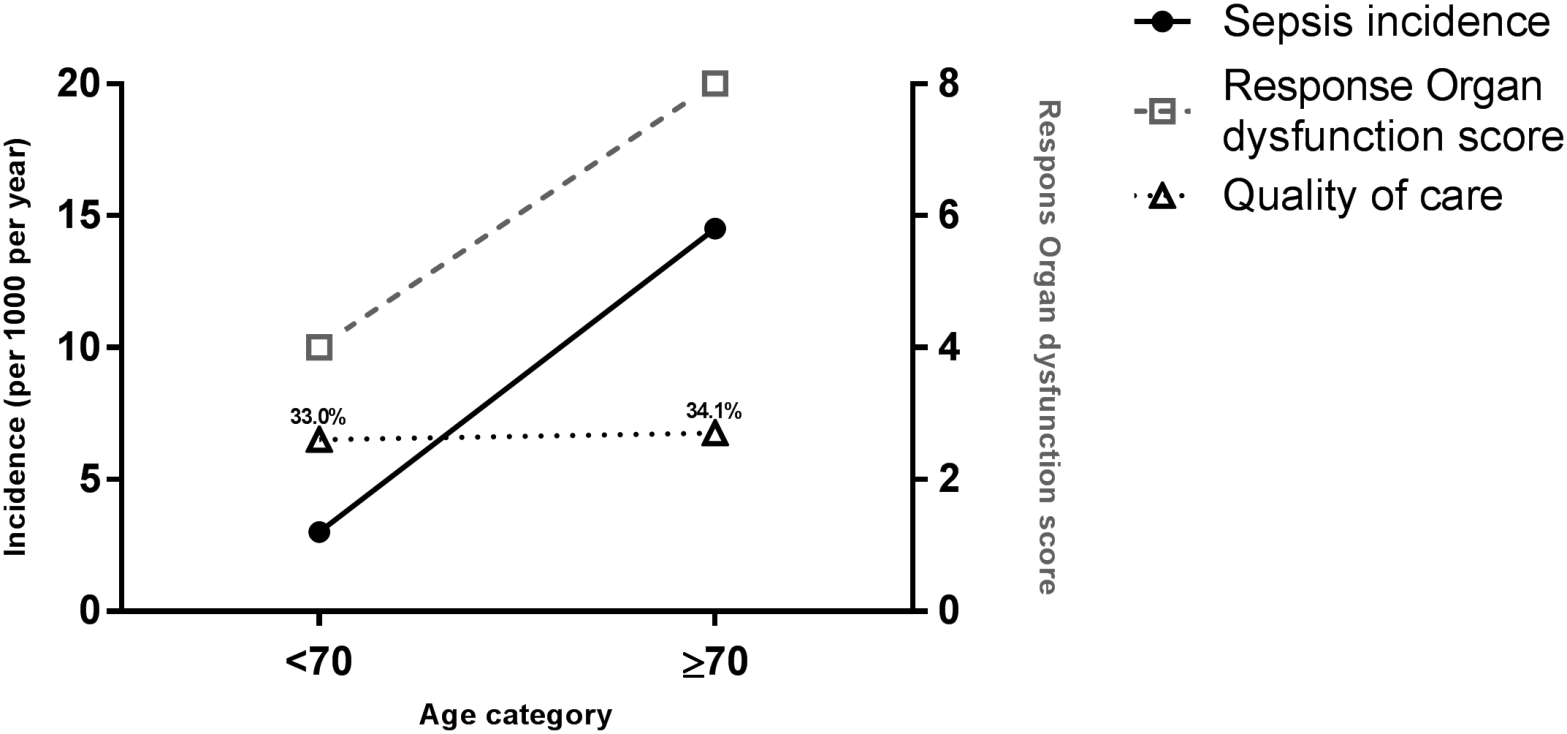
Illustration of the problem. With increasing age, both sepsis incidence and treatable organ dysfunction increase. However, quality of care, as assessed by full compliance to all nine quality performance measures, remains the same. *The increasing sepsis incidence, is according to an epidemiological study by Angus et al. [3]

The presence of twice as much potentially reversible organ dysfunction before ED treatment shows that even in older ED patients who are hospitalized with a suspected infection there is an opportunity to prevent mortality. However, if the goal of ED sepsis management of older patients is to reduce mortality, the percentage of patients in whom full compliance with all quality performance measures was achieved would need to be much higher. Unfortunately, the quality of care in terms of quality performance measures and SMR was similar in younger and older patients (Fig 2). This was in contrast to a study by Kakebeeke et al. [10] who showed that ED sepsis patients who were more ill were treated better, especially when they showed more clinically signs of organ dysfunction. Correspondent to our study, various studies with different study designs and settings, illustrated that care is worse in older patients [26–28].

An important finding of our study is that older patients present with more reversible acute organ dysfunction. We hypothesize that this is due to relatively late recognition of acute organ dysfunction by GPs or patients in the out ofhospital setting. This corresponds to previous studies in various populations which also found that recognition of illness severity was more difficult in older patients [2, 5, 9], which is presumably explained by atypical symptom presentation, like delirium, weakness, anorexia, malaise or falls, accompanied by a less explicit presentation of typical symptoms, like tachycardia, fever and hypotension[2, 5].

There are several explanations for the observation that quality of care is not better in older patients despite the fact that they enter the ED more ill than younger patients: firstly, because of poor recognition of acute onset organ dysfunction in older patients by physicians in the ED. We hypothesize that the lower heart rate and higher blood pressure in older patients are interpreted as “normal” while they are probably too low for adequate organ perfusion in older age, as indicated by the higher lactate, urea and creatinine (circulatory and renal failure), and higher incidence of altered mental status (inadequate brain perfusion; see Table 1). We doubt if the currently used sepsis management guidelines are equally effective in older patients compared to younger patients [2, 13]. Thehe RO components of the PIRO score may be less suitable for sepsis recognition by GPs and patients. Instead, better knowledge of baseline physiology in older patients would enable decision makers to better determine the significance of individual physiological parameters. For example, it is unlikely that 90 mmHg should be used as a cut-off to define shock in older patients [6]. It is likely that a higher threshold is more realistic in older patients who are known to need higher systolic blood pressures to maintain organ perfusion. Similarly, currently used threshold for respiratory rate and temperature may be insufficient to alert physicians of ongoing inadequate organ perfusion in the ED, resulting in a doubling of in-hospital mortality (Table 5).

**Table 5:** Quality of care as assessed by standardized mortality ratio (SMR) in the groups younger and older than 70 years.

Outcome	Age <70	Age ≥70	P-value
N (%)	1537 (64.9)	833 (35.1)	
Observed mortality, n (%) (20)	71 (4.6 (3.6–5.6))	77 (9.2 (7.4–11.5))	<0.001
Expected mortality (%) *	6.83 (5.57–8.09)	13.49 (11.17–15.81)	<0.001
SMR (Observed / expected mortality) (20)	0.68 (0.53–0.85)	0.69 (0.54–0.85)	>0.05

The SMR between patients of 70 years and older and patients younger than 70 years is similar.

Abbreviation: SMR = Standardized mortality ratio = observed divided by expected mortality. An SMR <1 indicates ED management is better than expected.

The number between brackets indicates the amount of missing variables.

* Expected mortality is calculated at ED entrance (before ED treatment) with the PIRO-score, by means of the derivation cohort and the two validation cohorts in the PIRO study by Howell et al [13].

Consequently, adequate fluid resuscitation is being withheld, often for unrealistic fear for fluid overload because of chronic cardiac co-morbidity.

This is in line with the findings of Churpek and colleagues who demonstrated that in ED patients older than 65 years, the modified early warning score and vital signs were less accurate for detection of cardiac arrest on a hospital ward compared to patients younger than 65 years old [9]. They suggested that age adjusted risk stratification is needed to prevent this devastating event. Likewise, severity scores used for ED sepsis patients, like PIRO, MEDS and qSOFA, might be less accurate in older patients. If sepsis recognition needs to be improved age adjusted risk stratification tools may be needed. The large difference in the presence of an altered mental status and DNR status in our study suggests that these variables could be used for early identification of sepsis in older patients with a suspected infection.

A second explanation for the lack of better ED treatment in older patients could be the underestimation of the older patients’ desire for aggressive treatment by the family and physician [27], and to the physicians low priority to live-sustaining treatment in older patients [28]. Interestingly, many older patients have a DNR status and do not wish to receive ICU treatment or mechanical ventilation. In those patients, prevention of co-morbidity and mortality depends solely on optimization of ED treatment.

Although our study has its strengths like the large unselected patient population and the large number of quality performance measures, there are several limitations.

First, there were some variables with many missings, like respiratory rate. However, the assumption that respiratory rate was normal (below 20/min) in case of an unregistered respiratory rate seems reasonable and has been done before in many studies. It is unlikely that this would have had a large impact on the calculations of the initial disease severity at ED presentation.

Second, ethical concerns prevented the performance of a randomized controlled trial. We therefore had to assess the impact of full compliance to all quality performance measures in an observational study which is more prone to selection bias and unmeasured confounding. We believe that the most important confounders, including demographics, co-morbidities, sources of infection, illness severity and aspects of ED management were accounted for in the analysis, mitigating the impact of bias on our study findings. More importantly, in a recently published study with a similar methodology we have done many sensitivity analyses to show that the impact of confounding is limited [8]. Finally, it is likely that the extent of confounding is similar between older and younger patients, so the conclusion that full compliance to all nine quality performance measures has impact on mortality still holds.

Finally, we realize that we did not take into account specific geriatric patients characteristics, like cognitive function which have been shown to affect patients outcomes, especially in older patients [29, 30]. However, previous studies demonstrated that there is a lack of recognition and registration of cognitive impairment in older patients by ED physicians [29, 31], thus in reality probably even more older patients presented with an altered mental status, strengthening our conclusion of poor recognition of organ failure in older patients.

In conclusion, the quality of care of older and younger ED sepsis patients is similar despite the presence of almost twice as much potentially reversible acute organ dysfunction in older patients at ED presentation. Our study suggests that acute sepsis-related organ dysfunction is recognized relatively late in the out ofhospital setting in older patients. Moreover, it suggests that acute organ dysfunction is poorly recognized in the ED, resulting in less aggressive ED management, with a higher, potentially avoidable, in-hospital mortality. Future studies should confirm our findings in a different database and investigate whether risk stratification tools for sepsis, like the newly developed qSOFA score adequately identifies older patients with high risk for sepsis.

## Supporting information

S1 Fig The sepsis screening algorithm used in the quality improvement program. (DOC)

S2 Fig Flow-diagram which was used to define appropriateness of the initial dose of antibiotics. (TIF)

S1 Tables Additional analyses investigating the association between PIRO score and full bundle compliance. (DOCX)

S2 Tables Additional multivariable regression analyses for in-hospital mortality in emergency department patients older and younger than 70 years. (DOCX)
