# Supplementary material for: Initial disease severity and quality of care of emergency department sepsis patients who are older or younger than 70 years of age
Source: PLoS One. 2017 Sep 25;12(9):e0185214. doi: 10.1371/journal.pone.0185214 (PMC5612649; doi:10.1371/journal.pone.0185214)
Supplement: S1 Fig — (DOC) [file pone.0185214.s001.doc]

Does the patient have a suspected infection and triage category yellow, orange or red?

**Nrse**

**Is there 1 alarming symptom or more potential alarming symtoms?**

**No**

**Re-evaluate patient regularly**

**Yes**

Inform physician and provide all vital signs

Give O_2_, give i.v. NaCl 0,9% 500 ml. Time:……..u.

Take 2 sets bloodcultures and other cultures if indicated. Time:……..u.

Lab: Leuco’s, Diff., Hb, thrombocytes, creatinine, urea, Na, K, ALAT, ASAT, ɣ-GT, AF,

bilirubin, amylase, CRP, lactate, troponin, INR/APTT. Consider blood gas.

Ask physician what antibiotics should be administered.

Place urinary catheter if blood pressure syst.< 90 mmHg, MAP < 65 mmHg (ask physician)

Give 1 to 1,5 liter NaCl 0,9% in 30 min.

Communicate suspected diagnosis with nurse

Consult infectious disease specialist if necessary

Check if all cultures are taken and if additional test

Are indicated.

Administer antibiotics directly based on focus

and institutional protocol. **Time:………….hrs**

Evaluate effect of treatment*

Evaluate labs

Search for signs of new onset organ failure**

**Therapy is not effective**

**Signs of organ failure**

**Consider**

**ICU consultation (pager 9182)**

**Admission ward**

**(Possible discharge home)**

Alarming symptoms

SO_2_ < 90%

Bloodpres. syst. < 90 mmHg

Bloodpres syst. > 40 mmHg gedaald

Skin signs/capillary refill > 2 sec.

Meningeal signs

Altered mental status

Petechiën

Potential alarming symptoms

Tachypnoe > 20/min

Tachycardia > 90/min

Temperature > 38 of < 36°C

Immuno-compromised

**POSSIBLE SEPSIS!**

Pneumonia (sputum culture)

Intra-abdominal infection

Urineweginfectie (urine culture)

Skin/bone/joint infection

Meningitis (liquor culture)

Implantant/line infection/TSS

Endocarditis

Other: i.e. malaria

SO_2_ > 90%, no signs of exhaustion

Bloodp. syst. > 90 mmHg, MAP > 65 mmHg. Time:… hr

Urine production > 0,5 ml/kg/hr

Normal mental status Tijd:….....u

**Remarks**:......................................................................

Respiratoiry insufficiency

Lactate > 4 mmol/L

Creatinine > 178 ųmol/L

INR >1,5 of APTT > 60 sec

Trombocytes < 100 10^9^/L

Bilirubin > 34 µmol/L

**Yes**

**Suspected**

**SEVERE SEPSIS!**

**F**

**O**

**C**

**U**

**S**

**Therapy is effective**

**No signs of organ failure**

**Nrse**

**Nrsee**

**Dr**

**Dr**

**Possible signs of new-onset organ failure****

**Targets for an effective therapy***
