# Supplementary figures and images for: Initial disease severity and quality of care of emergency department sepsis patients who are older or younger than 70 years of age
Source: PLoS One. 2017 Sep 25;12(9):e0185214. doi: 10.1371/journal.pone.0185214 (PMC5612649; doi:10.1371/journal.pone.0185214)

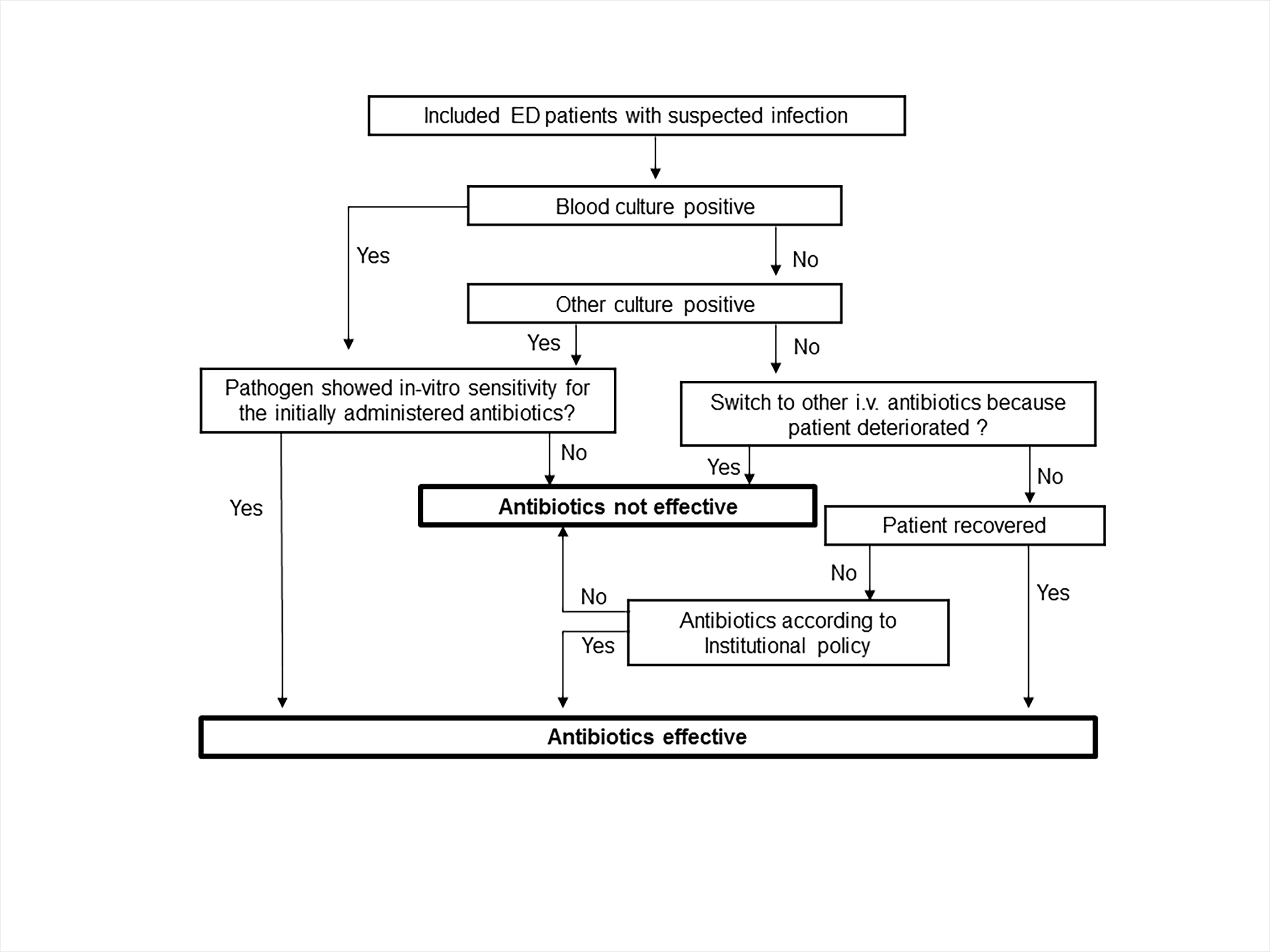

Supplement: S2 Fig — (TIF) [file pone.0185214.s002.tif]
