# Supplementary material for: Initial disease severity and quality of care of emergency department sepsis patients who are older or younger than 70 years of age
Source: PLoS One. 2017 Sep 25;12(9):e0185214. doi: 10.1371/journal.pone.0185214 (PMC5612649; doi:10.1371/journal.pone.0185214)
Supplement: S1 Tables — (DOCX) [file pone.0185214.s003.docx]

**S3 TABLES.**

**Additional analyses 1a.**

**ED patients >=70 years.**

PIRO score as continuous variable. Conclusion: No association between PIRO score as a measure of disease severity and full bundle compliance.

| **Variables in the Equation** | | | | | | | | | |
| --- | --- | --- | --- | --- | --- | --- | --- | --- | --- |
|  | | B | S.E. | Wald | df | Sig. | Exp(B) | 95% C.I.for EXP(B) | |
|  |  |  |  |  |  |  |  | Lower | Upper |
| Step 1^a^ | PIROSCORE | ,015 | ,016 | ,854 | 1 | ,355 | 1,015 | ,983 | 1,048 |
|  | Constant | -,734 | ,217 | 11,436 | 1 | ,001 | ,480 |  |  |
| a. Variable(s) entered on step 1: PIROSCORE. | | | | | | | | | |

PIRO score in three groups (0-8, 9-16, >17). Conclusion: No association between PIRO score as a measure of disease severity and full bundle compliance.

| **Variables in the Equation** | | | | | | | | | |
| --- | --- | --- | --- | --- | --- | --- | --- | --- | --- |
|  | | B | S.E. | Wald | df | Sig. | Exp(B) | 95% C.I.for EXP(B) | |
|  |  |  |  |  |  |  |  | Lower | Upper |
| Step 1^a^ | Piro_driegroepen |  |  | 2,550 | 2 | ,279 |  |  |  |
|  | Piro_driegroepen(1) | ,219 | ,190 | 1,331 | 1 | ,249 | 1,245 | ,858 | 1,805 |
|  | Piro_driegroepen(2) | -,089 | ,277 | ,104 | 1 | ,747 | ,915 | ,532 | 1,573 |
|  | Constant | -,684 | ,167 | 16,783 | 1 | ,000 | ,505 |  |  |
| a. Variable(s) entered on step 1: Piro_driegroepen. | | | | | | | | | |

**Additional analyses 1b.**

**ED patients < 70 years. Conclusion: In very ill ED patients younger than 70 years (PIRO score > 18) the odds for full compliance are lower (OR of 0.453) but this is not a significant OR since the overall P value is 0.067.**

PIRO score in three groups (0-8, 9-16, >17). Conclusion: No association between PIRO score as a measure of disease severity and full bundle compliance.

| **Variables in the Equation** | | | | | | | | | |
| --- | --- | --- | --- | --- | --- | --- | --- | --- | --- |
|  | | B | S.E. | Wald | df | Sig. | Exp(B) | 95% C.I.for EXP(B) | |
|  |  |  |  |  |  |  |  | Lower | Upper |
| Step 1^a^ | Piro_driegroepen |  |  | 5,402 | 2 | ,067 |  |  |  |
|  | Piro_driegroepen(1) | ,009 | ,113 | ,006 | 1 | ,939 | 1,009 | ,808 | 1,259 |
|  | Piro_driegroepen(2) | -,792 | ,346 | 5,231 | 1 | ,022 | ,453 | ,230 | ,893 |
|  | Constant | -,571 | ,075 | 57,402 | 1 | ,000 | ,565 |  |  |
| a. Variable(s) entered on step 1: Piro_driegroepen. | | | | | | | | | |

PIRO score as continuous variable. Conclusion: No association between PIRO score as a measure of disease severity and full bundle compliance.

| **Variables in the Equation** | | | | | | | | | |
| --- | --- | --- | --- | --- | --- | --- | --- | --- | --- |
|  | | B | S.E. | Wald | df | Sig. | Exp(B) | 95% C.I.for EXP(B) | |
|  |  |  |  |  |  |  |  | Lower | Upper |
| Step 1^a^ | PIROSCORE | -,017 | ,011 | 2,379 | 1 | ,123 | ,983 | ,962 | 1,005 |
|  | Constant | -,450 | ,108 | 17,396 | 1 | ,000 | ,638 |  |  |
| a. Variable(s) entered on step 1: PIROSCORE. | | | | | | | | | |
