# Supplementary material for: Initial disease severity and quality of care of emergency department sepsis patients who are older or younger than 70 years of age
Source: PLoS One. 2017 Sep 25;12(9):e0185214. doi: 10.1371/journal.pone.0185214 (PMC5612649; doi:10.1371/journal.pone.0185214)

**S4 TABLES.**

**Additional analyses 2a.** Multivariable logistic regression analysis for in-hospital mortality

**ED patients >= 70 years.**

| **Variables in the Equation** | | | | | | | | | |
| --- | --- | --- | --- | --- | --- | --- | --- | --- | --- |
|  | | B | S.E. | Wald | df | Sig. | Exp(B) | 95% C.I.for EXP(B) | |
|  |  |  |  |  |  |  |  | Lower | Upper |
| Step 1^a^ | Piro_driegroepen |  |  | 5,946 | 2 | ,051 |  |  |  |
|  | Piro_driegroepen(1) | ,195 | ,372 | ,273 | 1 | ,601 | 1,215 | ,585 | 2,521 |
|  | Piro_driegroepen(2) | ,925 | ,441 | 4,400 | 1 | ,036 | 2,522 | 1,063 | 5,987 |
|  | Full_bundle_compliance | -,529 | ,291 | 3,292 | 1 | ,070 | ,589 | ,333 | 1,043 |
|  | Opname_IC_MC | 1,507 | ,304 | 24,498 | 1 | ,000 | 4,511 | 2,484 | 8,191 |
|  | UMC_vs_Perifeer | -,191 | ,283 | ,453 | 1 | ,501 | ,826 | ,474 | 1,440 |
|  | Constant | -2,548 | ,405 | 39,608 | 1 | ,000 | ,078 |  |  |
| a. Variable(s) entered on step 1: Piro_driegroepen, Full_bundle_compliance, Opname_IC_MC, UMC_vs_Perifeer. | | | | | | | | | |

| **Hosmer and Lemeshow Test** | | | |
| --- | --- | --- | --- |
| Step | Chi-square | df | Sig. |
| 1 | 1,720 | 7 | ,974 |

| **Area Under the Curve** | | | | |
| --- | --- | --- | --- | --- |
| Test Result Variable(s): Predicted probability | | | | |
| Area | Std. Error^a^ | Asymptotic Sig.^b^ | Asymptotic 95% Confidence Interval | |
|  |  |  | Lower Bound | Upper Bound |
| ,684 | ,036 | ,000 | ,614 | ,754 |
| The test result variable(s): Predicted probability has at least one tie between the positive actual state group and the negative actual state group. Statistics may be biased. | | | | |
| a. Under the nonparametric assumption | | | | |
| b. Null hypothesis: true area = 0.5 | | | | |


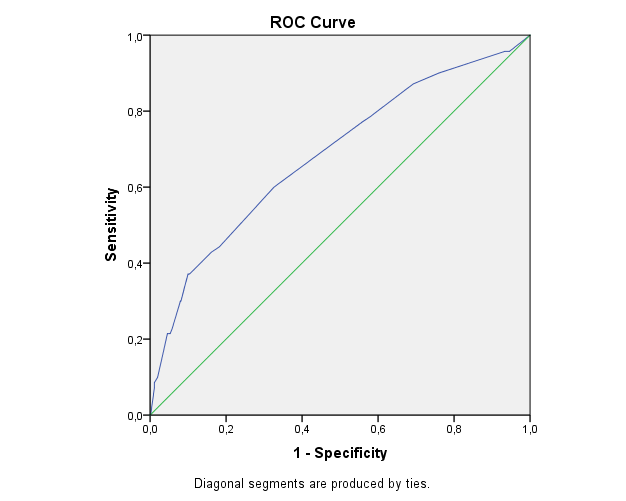


**Additional analyses 2a.** Multivariable logistic regression analysis for in-hospital mortality

**ED patients < 70 years.**

| **Variables in the Equation** | | | | | | | | | |
| --- | --- | --- | --- | --- | --- | --- | --- | --- | --- |
|  | | B | S.E. | Wald | df | Sig. | Exp(B) | 95% C.I.for EXP(B) | |
|  |  |  |  |  |  |  |  | Lower | Upper |
| Step 1^a^ | Piro_driegroepen |  |  | 39,119 | 2 | ,000 |  |  |  |
|  | Piro_driegroepen(1) | 1,595 | ,403 | 15,635 | 1 | ,000 | 4,930 | 2,236 | 10,872 |
|  | Piro_driegroepen(2) | 3,137 | ,503 | 38,925 | 1 | ,000 | 23,036 | 8,598 | 61,719 |
|  | Full_bundle_compliance | -,538 | ,325 | 2,735 | 1 | ,098 | ,584 | ,309 | 1,105 |
|  | Opname_IC_MC | 1,498 | ,305 | 24,179 | 1 | ,000 | 4,472 | 2,462 | 8,125 |
|  | UMC_vs_Perifeer | ,494 | ,372 | 1,765 | 1 | ,184 | 1,639 | ,791 | 3,396 |
|  | Constant | -4,919 | ,501 | 96,298 | 1 | ,000 | ,007 |  |  |
| a. Variable(s) entered on step 1: Piro_driegroepen, Full_bundle_compliance, Opname_IC_MC, UMC_vs_Perifeer. | | | | | | | | | |

| **Hosmer and Lemeshow Test** | | | |
| --- | --- | --- | --- |
| Step | Chi-square | df | Sig. |
| 1 | 3,019 | 6 | ,806 |

| **Area Under the Curve** | | | | |
| --- | --- | --- | --- | --- |
| Test Result Variable(s): Predicted probability | | | | |
| Area | Std. Error^a^ | Asymptotic Sig.^b^ | Asymptotic 95% Confidence Interval | |
|  |  |  | Lower Bound | Upper Bound |
| ,816 | ,027 | ,000 | ,763 | ,869 |
| The test result variable(s): Predicted probability has at least one tie between the positive actual state group and the negative actual state group. Statistics may be biased. | | | | |
| a. Under the nonparametric assumption | | | | |
| b. Null hypothesis: true area = 0.5 | | | | |


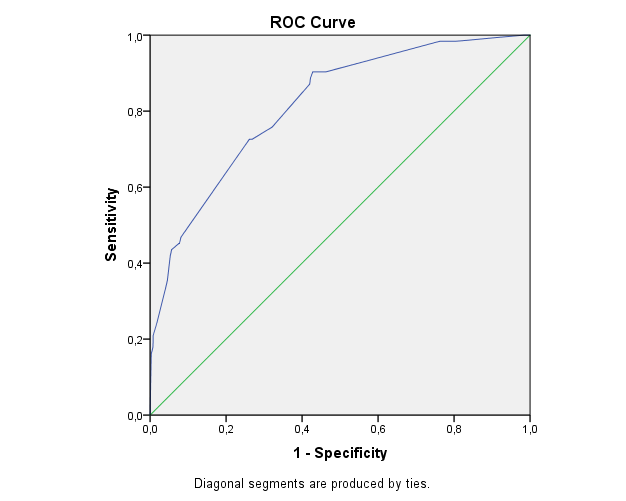

Supplement: S2 Tables — (DOCX) [file pone.0185214.s004.docx]
